# CO_2_ Photoreduction Under Visible Light by TiO_2_ and Carbon Dots Derived from Pyrolized Bio‐Oil

**DOI:** 10.1002/open.202400286

**Published:** 2024-11-26

**Authors:** Giorgia Ferraro, Marco Pizzolato, Teresa Botrè, Giuseppina Cerrato, Federica Menegazzo, Michela Signoretto

**Affiliations:** ^1^ Department of Molecular Sciences and Nanosystems Ca' Foscari University of Venice via Torino 155 30172 Venice Italy; ^2^ University of Turin via Pietro Giuria 7 10125 Torino Italy

## Abstract

Herein, we report a study on pyrolysis bio‐oil upgrading from leather shaving waste to dope in situ titania (TiO_2_) with carbon dots (cds). The cds doped TiO_2_ exhibits remarkable activity as photocatalyst under solar light for the direct conversion of carbon dioxide (CO_2_) and water vapor (H_2_O) to methane (CH_4_). Morover, the catalytic activity also increased under uv radiation.

## Graphical abstract

This communication demonstrates the possibility to efficiently exploit the liquid bio‐oil fraction from leather shaving waste to upcycle carbon dioxide. In fact, leather shaving waste bio‐oil is an effective bioproduct to prepare carbon dots that were used to dope titania. The new photocatalysts were effectively used in CO_2_ photoreduction under visible light.

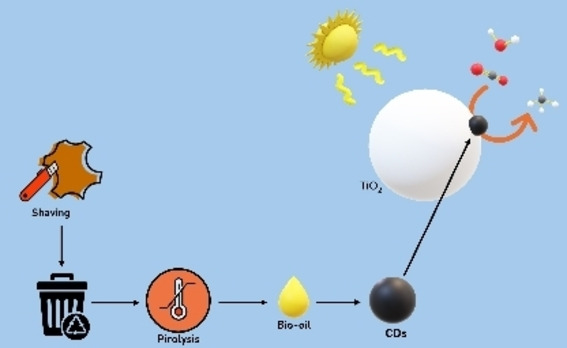

## Introduction

To meet the carbon neutrality standards set by the European Union, new strategies to produce fuels and chemicals are required. Photocatalysis is regarded as one of the future possible ways to catalyse industrial processes.^[1]^ This research topic is gaining an increasing attention since it allows to perform catalytic reactions under mild conditions exploiting solar radiation as energy source.^[2]^ The most studied reaction to photocatalytic upcycle carbon dioxide is its photoreduction. This has a great potential to meet the carbon‐neutrality standards which have been set by the European Union, allowing to convert a pollutant in added value chemicals using water as reducing agent. The products, such as methane and methanol, can be then directly used as fuels or energy vectors to feed the existing infrastructures.^[3]^ However, due to the intrinsic inertness of carbon dioxide, a strong energy input is required. Titanium dioxide (TiO_2_) is a wide studied semiconductor with a suitable bandgap to perform both CO_2_ reduction and water oxidation.^[4]^ However, titania can absorb only the UV‐fraction of the solar radiation, which makes up only 4 % of the solar spectrum, thus limiting its performance.^[5]^ Carbon dots (CDs) are zero‐dimensional carbon‐based materials with diameter below 10 nm.^[6]^ Such nanomaterials can be prepared according to two main strategies, either top‐down or bottom‐up. In this manuscript, a bottom‐up procedure, particularly a hydrothermal treatment, was chosen as a simple, environmentally friendly and cost‐effective method. Here, organic molecules undergo thermal decomposition forming the carbon nanoparticles.^[7]^ Recently, various starting materials and protocols have been investigated for their preparation, including biomass‐based materials. Among these, several studies have reported the use of renewable sources, such as lignocellulosic waste and algae biomass, as precursors for carbon dots, due to their availability in nature, as well as their reactivity and versatility.[^8^, ^9^, ^10^, ^11^, ^12^] These alternatives provide a sustainale approach for producing nanomaterials, reducing reliance on specific waste materials and improving the scalability of the process. Furthermore, the use of bio‐oil, i. e. the liquid fraction of biomass pyrolysis, as a starting material for carbon dots production has not been widely investigated, nor has it been extensively applied in the CO_2_ photoreduction. In this work tannins rich bio‐oil, derived from leather shaving waste, has been chosen as raw material to obtain N‐rich CDs. Leather tannery waste has not yet been studied in literature for this application: it is a second‐generation animal biomass, deriving from a “metal free” tanning process. This waste is generated during the mechanical shaving process of tanned leather, which corresponds to 25 % by weight of tanned leather and therefore represents a large amount of waste, being projected to increase. Leather is mainly composed of collagen in a 50–70 % by weight.^[13]^ Collagen pyrolysis produce a N‐rich bio‐oil and hence N‐rich carbon dots can be prepared, which are known for their excellent electron transfer ability upon irradiation making them excellent candidates for photocatalysis.[^14^, ^15^] Few papers have been published, reporting about testing CDs for CO_2_ reduction to methanol, methane, or formic acid.[^16^, ^17^, ^18^, ^19^] Photoelectric properties of CDs must meet specific values to be effective for the reaction, in terms of band‐gap and light absorption properties as well. The best results with these photocatalytic materials (CDs) have been obtained by Wang *et al*. who were able to selectively convert CO_2_ to methanol.[^19^, ^20^] Hence, to the best of our knowledge, this is the first time that these types of materials are investigated for this target application which could be promising and requires more study and optimization. Particularly, the aim of this work is to upcycle the pyrolysis bio‐oil from leather shaving waste^[21]^ to obtain a cheap and active photocatalyst for CO_2_ photoreduction.

## Experimental

**Figure 1:**
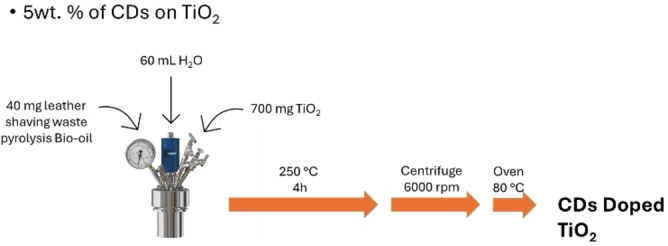
Synthetic procedure for CDs doped TiO_2._

TiO_2_‐CDs photocatalyst was prepared from commercial titania (P25) and CDs were obtained *in situ* from leather shaving waste derived pyrolysis^[22]^ bio‐oil according to the procedure reported in Figure 1.

## Results and Discussion

**Figure 2:**
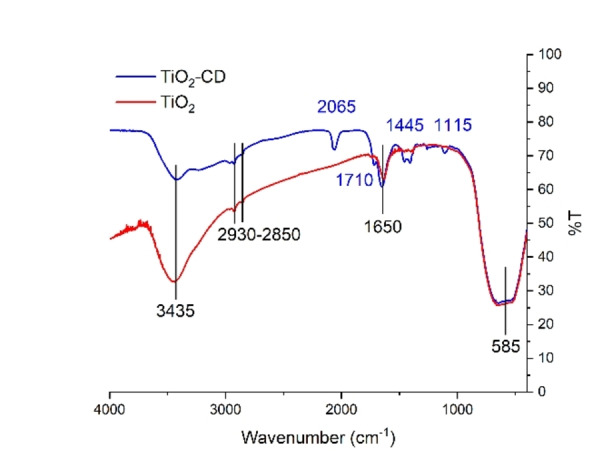
FT‐IR spectra of TiO_2_ and TiO_2_‐CD.

After materials synthesis, characterizations have been carried out. In Figure 2 FT‐IR spectra of the CDs promoted TiO_2_ sample compared with bare TiO_2_ are reported. Both spectra some of the peculiar spectral components of TiO_2_ are present, such as the band located at slightly above 3400 cm^−1^ (due to the stretching mode of all −OH groups present stretching, whose spectroscopic counterpart due to the bending mode lies at ca. 1630 cm^−1^), besides a net component located at 580 cm^−1^ and due to the Ti−O stretching mode.^[23]^ In the promoted sample some more characteristic peaks are present, due to the presence of carbon dots. In particular, the signal at 2065 cm^−1^ refers to the C≡C stretching mode, the signal at 1710 cm^−1^ is relative to the C=O stretching of aldeydic groups on the surface, the signals at 1445 cm^−1^ refers to C−N stretching mode and to methyl and methylene groups on CDs surace, and the signal at 1115 cm^−1^ refers to C−N amines stretching mode: altogether, the presence of all these spectral components confirms the presence of CDs doping on titania.

**Figure 3:**
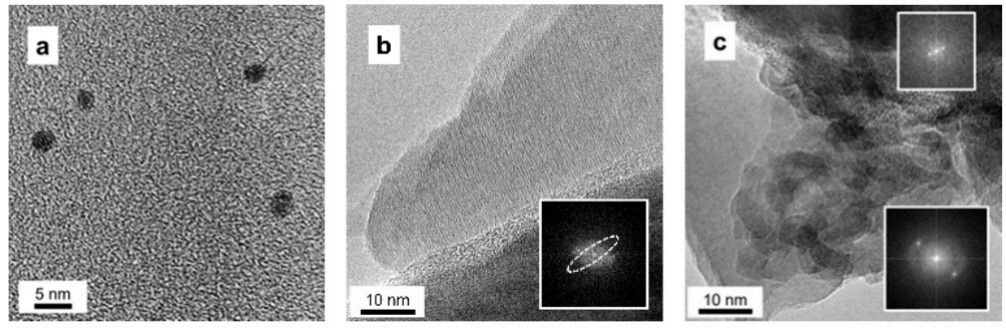
HR‐TEM images of the TiO_2_‐CD sample.

HR‐TEM images reveal the presence of carbon dots with a size of ca. 3 nm (Figure 3a). Moreover, from the analysis of the simulated diffraction pattern (obtained by the FFT elaboration of the direct images), it is possible to single out a general graphitic coating on top of the TiO_2_ nanoparticles (Figures 3b and c). The relevant diffraction planes are those typical of graphite, and namely (003), (006), (009) crystalline planes, according to the ICDD card n. 01–074‐2328. It is then preliminary possible to state that the synthetic procedure used is an effective and simple approach to produce CDs from pyrolysis bio‐oil.

**Figure 4:**
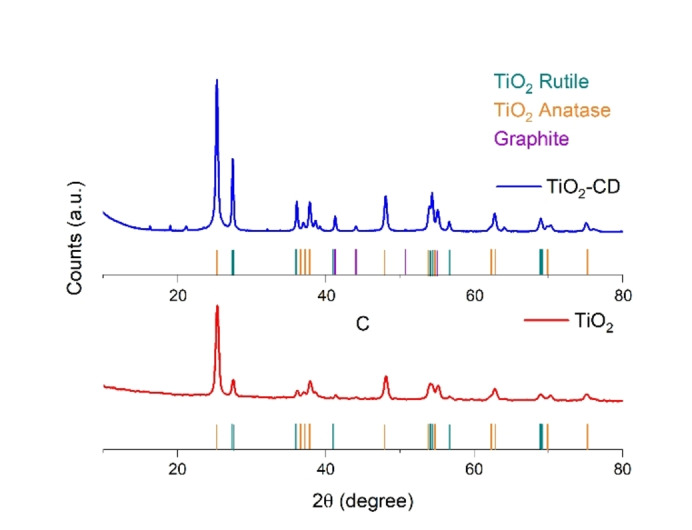
XRD pattern of TiO_2_ and TiO_2_‐CD.

The XRD profile of the promoted sample (Figure 4) resembles the typical signals of rutile and anatase phase of titanium dioxide, as per the comparison with the P25 XRD profile: however, it is possible to note also other peaks which can be ascribed to the graphitic core of CDs [24]. Hence, XRD analysis further confirms the presence of a carbon dots doping on photocatalyst surface in accordance with both HR‐TEM and FT‐IR results.

**Figure 5:**
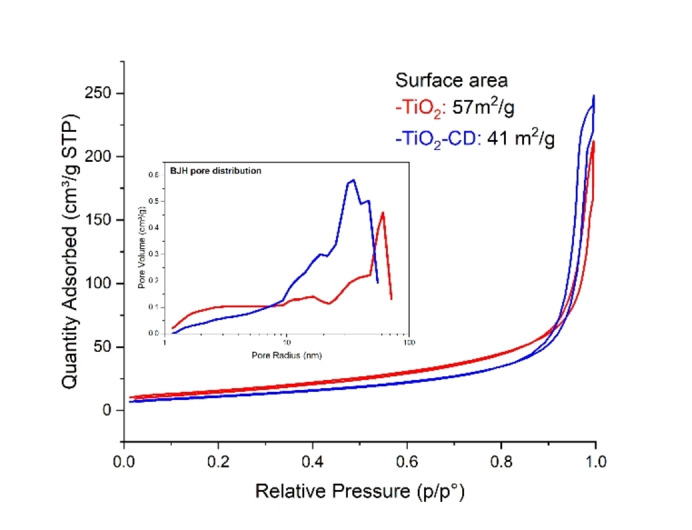
N_2_ physisorption of TiO_2_ and TiO_2_‐CD.

N_2_ physisorption analyses were performed to evaluate if carbon dots addition has both influenced and altered TiO_2_ pore structure. The isotherms reported in Figure 5 evidenced that carbon addition has decreased the surface area from 57 to 41 m^2^/g. Moreover, the BJH pore distribution indicates that the promoted sample presents a higher mesoporosity due to CDs presence. This confirms that carbon dots have preferentially occupied titania macropores thus generating a mesopore pattern.

**Figure 6:**
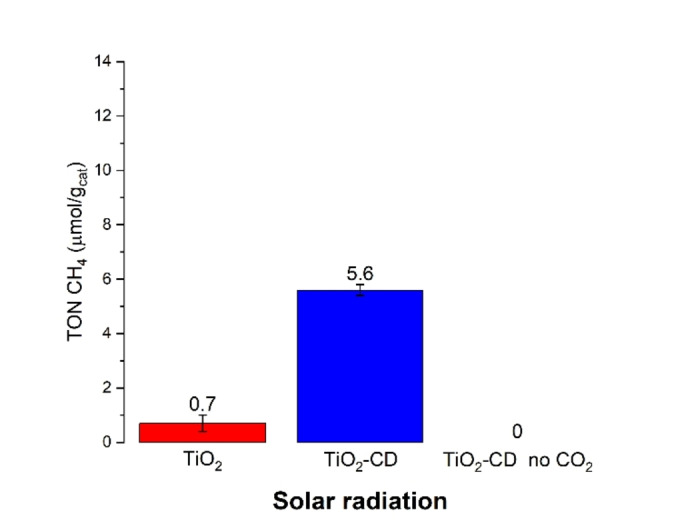
Activity test of TiO_2_ and TiO_2_‐CD under solar radiation.

Gas‐phase CO_2_ photoreduction to CH_4_ showed an increase in activity after CDs introduction under solar radiation (Figure 6). In particular, the 5‐fold increase under solar light can be ascribed to the synergistic interaction between CDs and TiO_2_. To exclude the possibility of false positive methane formation due to carbon dots degradation, a test with He instead of CO_2_ was performed: no methane formation was observed, as expected, thus confirming that methane production is derived from CO_2_ reduction.

**Figure 7:**
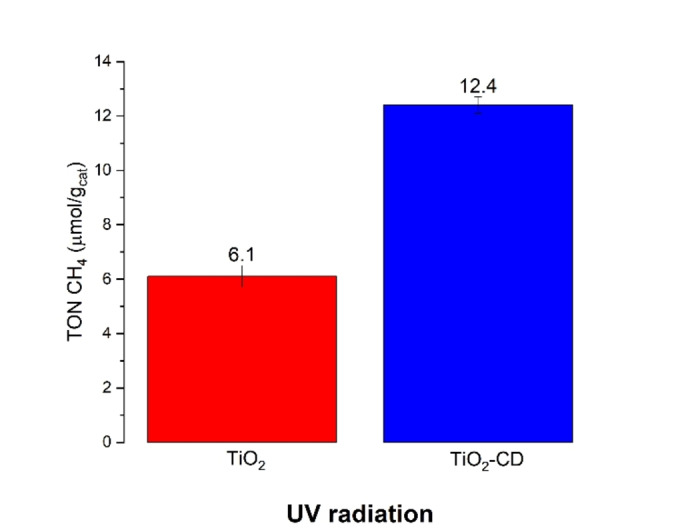
Activity test of TiO_2_ and TiO_2_‐CD under UV radiation.

Even better results were obtained with CDs doping also under UV radiation (Figure 7). The activity increment was around two times if compared with the undoped sample. It is possible that the presence of CD‐TiO_2_ heterojunction helped in separating the photogenerated charges increasing lifetime and availability of excited electrons for the reaction.^[25]^

**Figure 8:**
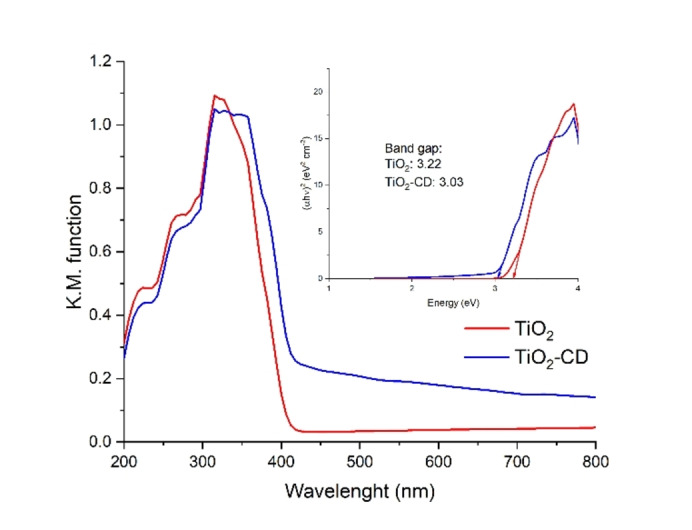
DRS spectra and Tauc Plot of TiO_2_ and TiO_2_‐CD.

To better investigate the reason of these results, we have performed also DRS analyses of both TiO_2_ and TiO_2_‐CD samples (Figure 8). The promoted sample exhibits a broadening in absorption in the visible region starting from 350 up to 800 nm. This can be ascribed to the presence of carbon dots which have improved the optical properties of the oxide. In particular, TiO_2_ is well known as photocatalyst, but it can only absorb UV light, which represents only the 4 % of the solar radiation. The doping strategy has hence increased the absorption range of the bare titania allowing to use a higher number of photons to generate excitons. The synergic effect can also be seen in the Tauc Plot, in which the reduced band gap from 3.22 to 3.03 eV is representative of the broader absorption in the visible region of the spectrum. These two aspects justify the observed increment in activity under solar light radiation. Therefore, CDs can act as a co‐catalyst absorbing photons and generating excitons, which in turn can be readily transferred to the titanium dioxide to perform the reaction.

The use of renewable raw materials, solar light and CO_2_ valorisation is part of a 360° circular perspective in line with the 2030 EU Agenda and the requirements of the current energy transition. The solid pyrolysis product from this biomass has already found promising applications.^[22]^

## Conclusions

This communication demonstrated the possibility to efficiently exploit the liquid bio‐oil fraction from leather shaving waste pyrolysis to upcycle CO_2_. The synthetic procedure used is very simple and can be easily scaled‐up, making this new type of material interesting also from the industrial point of view. This process is not only limited to the leather tannery waste biomass but could also be extended to other types of biomasses, including lignocellulosic, vegetal and animal sources, aligning with the principles of the circular economy. The carbon dots’ introduction has demonstrated to lower the band‐gap forming heterostructures which are efficient enhancers of titanium dioxide photocatalytic properties. In fact, the catalyst activity under solar radiation was increased by a 5‐fold factor. Moreover, an increased activity was observed also under UV radiation with an almost doubled activity.

## Author Contributions

Giorgia Ferraro: Investigator, Conceptualization; Marco Pizzolato: Conceptualization, Writing ‐ Original Draft; Teresa Botrè: Investigator; Giuseppina Cerrato: Investigator; Federica Menegazzo: Supervision, Writing ‐ Review & Editing; Michela Signoretto: Funding acquisition, Supervision, Writing ‐ Review & Editing.

## Conflict of Interests

The authors declare no conflict of interest.

1

## Data Availability

The data that support the findings of this study are available from the corresponding author upon reasonable request.
